# A systematic review of quality of life research in medicine and health sciences

**DOI:** 10.1007/s11136-019-02214-9

**Published:** 2019-06-11

**Authors:** K. Haraldstad, A. Wahl, R. Andenæs, J. R. Andersen, M. H. Andersen, E. Beisland, C. R. Borge, E. Engebretsen, M. Eisemann, L. Halvorsrud, T. A. Hanssen, A. Haugstvedt, T. Haugland, V. A. Johansen, M. H. Larsen, L. Løvereide, B. Løyland, L. G. Kvarme, P. Moons, T. M. Norekvål, L. Ribu, G. E. Rohde, K. H. Urstad, S. Helseth, the LIVSFORSK network

**Affiliations:** 1 Faculty of Health- and Sport Sciences, University of Agder, P.O Box 422, 4604 Kristiansand, Norway; 2 Department of Health Sciences, University of Oslo, P.O. Box 1084, Blindern, 0317 Oslo, Norway; 3 Faculty of Health, OsloMet – Oslo Metropolitan University, St. Olavs plass, P.O. Box 4, 0130 Oslo, Norway; 4 Department of Health and Caring Sciences, Western Norway University of Applied Sciences (HVL), P.O. Box 7030, 5020 Bergen, Norway; 5 Faculty of Health Sciences, UiT The Arctic University of Norway, P.O. Box 6050, Langnes, Tromsø, Norway; 6 University Hospital of North Norway, P.O. Box 100, 9038 Tromsø, Norway; 7 Faculty of Health Studies, VID Specialized University, Mailbox 184, Vinderen, NO 0319 Norway; 8 Department of Public Health and Primary Care, University of Leuven, P.O. Box 7001, Kapucijnenvoer, 3000 Leuven, Belgium; 9 Haukeland University Hospital, P.O. Box 1400, 5021 Bergen, Norway; 10 Faculty of Health Sciences, University of Stavanger, P.O. Box 8600, Forus, Norway; 11 Department of Clinical Research, SSHF, P.O. Box 416, 4604 Kristiansand, Norway

**Keywords:** Quality of life, Health-related quality of life, Systematic review

## Abstract

**Purpose:**

Quality of life (QOL) is an important concept in the field of health and medicine. QOL is a complex concept that is interpreted and defined differently within and between disciplines, including the fields of health and medicine. The aims of this study were to systematically review the literature on QOL in medicine and health research and to describe the country of origin, target groups, instruments, design, and conceptual issues.

**Methods:**

A systematic review was conducted to identify research studies on QOL and health-related quality of life (HRQOL). The databases Scopus, which includes Embase and MEDLINE, CINAHL, and PsycINFO were searched for articles published during one random week in November 2016. The ten predefined criteria of Gill and Feinstein were used to evaluate the conceptual and methodological rigor.

**Results:**

QOL research is international and involves a variety of target groups, research designs, and QOL measures. According to the criteria of Gill and Feinstein, the results show that only 13% provided a definition of QOL, 6% distinguished QOL from HRQOL. The most frequently fulfilled criteria were: (i) stating the domains of QOL to be measured; (ii) giving a reason for choosing the instruments used; and (iii) aggregating the results from multiple items.

**Conclusion:**

QOL is an important endpoint in medical and health research, and QOL research involves a variety of patient groups and different research designs. Based on the current evaluation of the methodological and conceptual clarity of QOL research, we conclude that the majority QOL studies in health and medicine have conceptual and methodological challenges.

**Electronic supplementary material:**

The online version of this article (doi:10.1007/s11136-019-02214-9) contains supplementary material, which is available to authorized users.

## Introduction

Quality of life (QOL) has become established as a significant concept and target for research and practice in the fields of health and medicine [1]. Traditionally, biomedical and not QOL outcomes have been the principal endpoints in medical and health research. However, during the past decades, more research has focused on patients’ QOL, and the use of QOL assessments has increased [2].

Understanding QOL is important for improving symptom relief, care, and rehabilitation of patients. Problems revealed by patients’ self-reported QOL may lead to modifications and improvement in treatment and care or may show that some therapies offer little benefit. QOL is also used to identify the range of problems that can affect patients. This kind of information can be communicated to future patients to help them anticipate and understand the consequences of their illness and its treatment. In addition, cured patients and long-term survivors may have continuing problems long after their treatment is completed. These late problems may be overlooked without QOL assessment. QOL is also important for medical decision-making because QOL is a predictor of treatment success and is therefore of prognostic importance. For instance, QOL has been shown to be a strong predictor of survival [1]. This prognostic ability suggests that there is a need for routine assessment of QOL in clinical trials [1].

Despite the importance of QOL in health and medicine, there is a continuing conceptual and methodological debate about the meaning of QOL and about what should be measured. There is no uniform definition of the concept; however, The World Health Organization (WHO) outlines one definition of QOL; “An individual’s perception of their position in the in the life in the context of the culture in which they live and in relation to their goals, expectations, standards and concerns” [3].

Moreover, the term health-related quality of life (HRQOL) is often described as: “A term referring to the health aspects of quality of life, generally considered to reflect the impact of disease and treatment on disability and daily functioning; it has also been considered to reflect the impact of perceived health on an individual’s ability to live a fulfilling life. However, more specifically HRQOL is a measure of the value assigned to duration of life as modified by impairments, functional states, perceptions and opportunities, as influenced by disease, injury, treatment and policy” [4].

QOL is a complex concept that is interpreted and defined in a number of ways within and between various disciplines. As a consequence, many different instruments are now used to assess QOL. These instruments were developed based mainly on empirical considerations and have not been developed from a definition or a conceptual model. Consequently, there is a lack of conceptual clarity about what QOL means and measures, which may pose a threat to the validity of QOL research [1].

Several conceptual and methodological analyses of QOL have been published [1, 5–8]. For instance, with the aim of determining the range of conceptual and methodological rigor of studies and of identifying temporal trends, Bratt and Moons [7] conducted a systematic literature review of all empirical studies of QOL in patients with congenital heart disease published since 1974. They applied ten review criteria that had been previously developed by Gill and Feinstein in 1994 [5] and further refined by Moons et al. in 2004 [8]. Bratt and Moons found slight but nonsignificant temporal improvements in conceptual and methodological rigor and in the use of assessment methods. However, most of the papers had substantial conceptual and methodological deficits. Despite 40 years of research on QOL in people with congenital heart disease, the review identified the prevalence of major weaknesses in the methodological rigor. We reasoned that this might also be the case in research on QOL in general medical and health research. Therefore, the aim of the present study was to perform a systematic review of QOL research in the fields of medicine and health, and to describe the country of origin, target groups, instruments, design, and conceptual issues in the current research.

## Methods

The review was designed as a systematic review with a short time frame, which was limited to one random week (a “snapshot”). Because a high number of QOL articles are published every year, it is not possible to review all. Therefore, a random selection can give a good picture of QOL research. We used the PRISMA (Preferred Reporting Items for Systematic Reviews and Meta-Analyses Statement) checklist to ensure rigor in conducting and the reporting of this systematic review [7]. The checklist comprises 27 items including those deemed essential for transparent reporting of systematic reviews. To evaluate the conceptual and methodological rigor, we used the same ten predefined criteria developed by Gill and Feinstein [5] and refined by Moons et al. [8].

### Data search

Systematic literature searches for publications referring to QOL or health-related quality of life (HRQOL) were conducted in collaboration with a trained librarian. To ensure broad coverage, the search term used was “*Quality of life* OR *Health*-*related quality of life*.” We searched for publications published during a randomly chosen week from November 19–26, 2016. The actual search was performed on November 26, and we searched for “the last 7 days” in the databases Scopus, which covers Embase and MEDLINE, CINAHL, and PsycINFO. The Scopus database allowed us to search for specific dates. The search resulted in 364 publications. To ensure that this week was not unique in terms of the number of articles published, we performed the same search strategy using the same databases for a random week 2 months later, in January 2017, which yielded a similar number of publications (*n* = 383).

### Eligibility criteria

The inclusion and exclusion criteria were developed a priori. A data extraction form was created before the review to identify the key characteristics of studies that met the criteria for inclusion. The main inclusion criteria were that QOL or HRQOL should be mentioned in the title or abstract and that the included studies should be peer-reviewed original research publications. The exclusion criteria were: conference abstract, non-English publication, editorial, opinion article, scientific statement, guideline, protocol, or review article.

### Data selection process

**Fig. 1:**
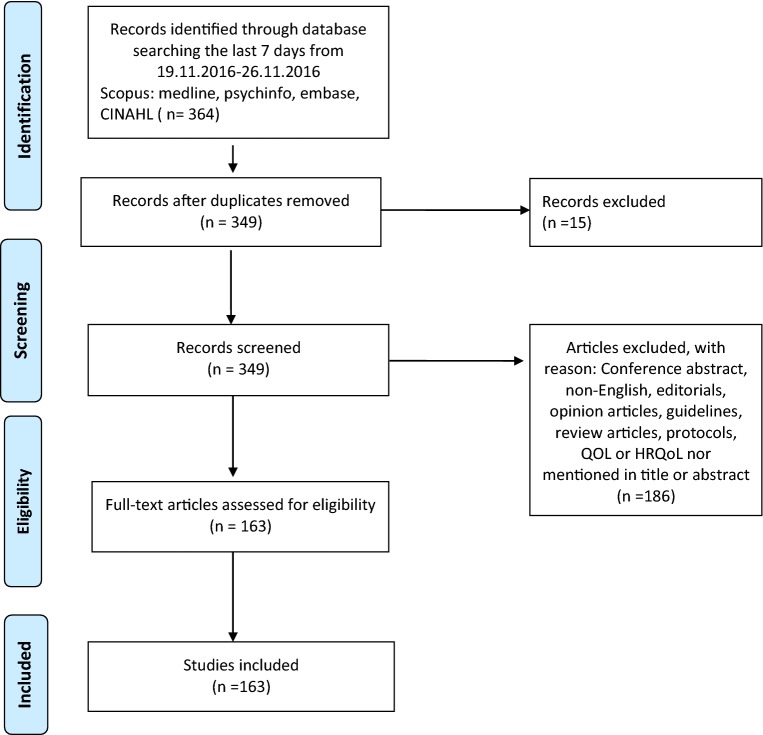
Flow chart of inclusion. Source: Reproduced From Moher D, Liberati A, Tetzlaff J, Altman DG, The PRISMA Group (2009). Preferred Reporting Items for Systematic Reviews and Meta- Analyses: The PRISMA Statement. PLoS Med 6(7): e1000097. 10.1371/journal.pmed1000097. For more information, visit https://www.prisma-statement.org.

The literature searches resulted in 364 publications. After removing duplicates, 349 papers were eligible for screening. Twenty-four QOL researchers participated in the screening process, and all papers were screened independently by title and abstract by two reviewers, who worked in pairs. In total, 186 publications were excluded during the screening process. The remaining 163 publications were included, read in full, and then independently reviewed and scored by the two reviewers before agreeing in a consensus meeting. In case of disagreement, consensus was achieved by three main investigators, one of whom was involved in the original review. A flowchart detailing the study selection and inclusion is shown in Fig. 1 (An online supplement with all references is included in the appendix).

Data extraction forms to register the key characteristics of the studies were used, and the following variables were registered: country, study design, number of participants, age groups (children or adults), and QOL instrument(s) used.

### Review criteria

In accordance with the aim of the study, we reviewed the included QOL publications in terms of country, study design, number of participants, age groups (children or adults), and QOL instrument(s) used. In addition, we reviewed the publications regarding how they dealt with conceptual issues and methodology [6] according to the criteria presented in Table 5.

## Results

### Description of QOL publications

#### Search results

The studies included in this review all used QOL and/or HRQOL as a concept. Of the included studies, 60 were from Europe and had been conducted in 17 different European countries. The Netherlands had the most with nine studies, and Spain and Germany had eight studies each; 47 studies were from North America (USA and Canada), and 41 were from Asian countries (Table 1).

**Table 1:** Country where the study was conducted

Europe	N
Netherlands	9
Germany	8
England, Spain,	7,7
Turkey, Italy, France	5,5,5
Slovenia	3
Portugal, Poland	2,2
Norway, Ireland, Switzerland, Denmark, Romania, Belgium, Croatia	1,1,1,1,1,1,1
North America	
USA	43
Canada	4
Asia	
China	18
Korea	5
India, Japan	4, 4
Iran, Indonesia, Pakistan, Taiwan	3,2,2
Singapore, Israel, Taiwan	1,1,1
Oceania	
Australia	7
South America	
Brasil	5
Africa	
Uganda, Nigeria, South-Africa	1,1,1

Sixty-one (38%) of the included studies had an experimental design involving either a randomized controlled trial (RCT) design or a quasi-experimental design. Fifty studies had a cross-sectional or descriptive design, and 37 had a cohort or longitudinal design. Six of the studies had a case-control design, seven studies were methodological or validation studies, one study had a qualitative design, and one study had a mixed-methods design (Table 2).

**Table 2:** Study design

Design	*N* (%)
RCT/experimental	61 (37.6)
Cross-sectional/descriptive	50 (31.6)
Cohort/prospective/longitudinal	37 (22.1)
Methodological	7 (4.1)
Case–control	6 (3.6)
Mixed methods	1 (0.6)
Qualitative	1 (0.6)

In 20 of the studies, the sample was children and/or adolescents. The other 143 studies included adults. The most prevalent patient groups studied were those with cancer (34 studies), mental illness (12 studies), heart disease (11 studies), gastrointestinal disease (11 studies), and chronic obstructive pulmonary disease (COPD) or asthma (seven studies). Seven studies included community samples or normal populations, and seven studies included older adults (Table 3).

**Table 3:** Number of studies related to patient groups, *N* = 163

Patient groups	Adults	Children	Total
Cancer	32	2	34
Mental illness	11	1	12
Heart disease	11	–	11
Gastrointestinal disease	10	1	11
Kidney/renal	9	1	10
Community sample/normal population	6	1	7
Chronic obstructive pulmonary disease/asthma	7	–	7
Orthopedic	6	2	7
Elderly	7	–	7
Hemophilia	4	1	5
Gynecological disease	4	–	4
Obesity	3	2	5
Pain	4	–	4
Epilepsy/cerebral paresis	2	2	4
Multiple sclerosis	4	–	4
Diabetes	2	1	3
Psoriasis	3	–	3
Developmental problems	–	2	2
Allergy			2
Cystic fibrosis	1	1	2
Eye disease	2		2
Fibromyalgia	2		2
Cosmetics	2	–	2
Tinnitus, oral health, trans gender, hearing loss, HIV, myositis, nasal septum, brain injury, eating disorder	1,1,1,1,1,1 1,1,1,1		10
Cleft lip		1	1
Myelomeningocele		1	1
Preterm		1	1

The 163 papers reviewed used 51 different questionnaires, which were both generic and disease specific. Generic QOL questionnaires were used in 66 of the studies of adults. The generic instruments most commonly used were the Short Form-36 (SF-36), EQ 5D, EORTC QLQ C-30, WHOQOL-BREF, and SF-12. Child-specific instruments were used in most of the studies on children, although four studies used questionnaires for adults. Of the child-specific instruments used, 12 were generic and four were disease specific. The PedsQL was used most frequently. An overview of the instruments used is given in Table 4.

**Table 4:** Quality of life instruments used (*N* = 163)

Instruments	Number of studies
Generic	
Short form SF-36	21
EQ-5D	16
WHOQOL-BREF	7
Short form SF-12	5
Cantrills ladder	2
Satisfaction with Life Scale (SWLS)	2
Disease specific	
EORTC QLQ C-30	15
Gastrointestinal QOL index (GIQLI)	4
Asthma Quality of Life Questionnaire (AQLQ)	3
Dermatology Life Quality score, (DLQI)	3
Stroke Specific QOL Scale	2
McGill QOL Questionnaire	2
The Haemo-QOL Questionnaire	2
Patient outcome measurement information system (PROMIS)	2
FACT-L	2
National Eye Institute Visual Functioning Questionnaire (NEI-VFQ-25)	1
Sexual Function Questionnaire-12 (PISQ-12)	1
DLQ1	1
QLESQ-SF	1
Melasma QOL questionnaire	1
Owestry dis index (ODI)	1
Inflam. Bowel Disease Questionnaire (IBDQ)	1
MG-QOL 15	1
NOSE nasal obstruction symptom evaluation	1
The ten-item Lehman’s quality of life (QOL) measure	1
Celiac dietary, CD quality of life	1
Epilepsy and Learning Disabilities Quality of Life Scale (ELDQOL)	1
Nutri- QOL	1
The Hand-Foot Skin Reaction QOL Questionnaire (HF-QOL-K)	1
Food Allergy Quality of Life Parental Burden	1
Seattle Angina Questionnaire (SAQ-QOL)	1
FertiQOL	1
QOL Alzheimer’s Disease Scale	1
Patient Health Questionnaire-2 score (PHQ2)	1
Incontinence Impact Questionnaire Short Form (31Q, IIQ-7)	1
Glaucoma-specific preference-based HRQOL instrument	1
CAS 20	1
CASP-12	1
Dartmount coopertive functional assessment charts (COOP)	1
Stoma-QOL Questionnaire	1
Kansas City Cardiomyopathy Questionnaire (KCCQ)	1
Children	
Generic	
PedsQl	5
Child Health Questionnaire (CHQ)	2
KINDL	2
KIDSCREEN 27	1
DISABKIDS HRQOL	1
Preschool Children’s Quality of Life Questionnaire (TAPQOL)	1
Disease specific	
PedsFact-BrS	1
Questionnaire QOL fécal Continence in Children (QQVCFCA)	1
Child oral health impact profile-(COHIP)	1
Cystic Fibrosis QOL Questionnaire-Revised	1

**Table 5:** Evaluation of methodological and conceptual rigor according to the criteria from Gill and Feinstein (*N *= 163)

Criteria	*N*	%
1. Did the investigator give a definition of quality of life?	22	13
2. Did the investigators state the domains they will measure as components of quality of life?	57	34
3. Did the investigators give reasons for choosing the instrument they used?	41	25
4. Did the investigator aggregate results from multiple items, domains or instruments into a single composite score for quality of life?	88	53
5. Were patients asked to give their own global rating for quality of life?	16	9
6. Was overall quality of life distinguished from health-related quality of life?	11	6
7. Were the patients invited to supplement the items listed in the instruments offered by the investigators that they considered relevant for their quality of life?	0	
8. If so, were these supplemental items incorporated into the final rating?	0	
9. Were patients allowed to indicate which items were personally important to them?	1	0.6
10. If so, were the importance ratings incorporated into the final rating?	0	

#### Evaluation according to the criteria

The evaluation of methodological and conceptual quality or rigor according to the criteria of Gill and Feinstein [5, 8] (Table 5) revealed that 22 (13%) of the 163 studies provided a definition of the concept QOL (criterion 1). In 57 of the papers (35%), the investigators stated the domains they measured as part of QOL (criterion 2). In 41 of the papers (25%), the investigators gave a specific reason for the choice of instrument to measure QOL (criterion 3). In 88 (53%) of the studies, the investigators had aggregated results from multiple items, domains, or instruments into a single composite score for QOL (criterion 4). However, few studies (9%) fulfilled criterion 5, concerning whether patients were asked to give their own global rating of QOL by a single item at the end of the questionnaire.

For criterion 6, in 11 (6%) of the included articles, QOL was distinguished from HRQOL. Evaluation of the studies showed that criteria 7–10 were not fulfilled; none of the studies provided an option for the participants to select additional items that are important to them. However, in one study, the respondents could indicate which of the given items are personally important to them, but the importance rates were not incorporated into the overall score.

## Discussion

The findings of this systematic snapshot review show that QOL research is truly international, involves a variety of target groups, and uses different research designs and many types of QOL measures. Moreover, few of the included studies provided a definition of the concept of QOL, and most articles had a low-quality score according to the criteria of Gill and Feinstein [5, 8].

However, some trends were apparent. Studies of QOL have been conducted in all parts of the world, but the USA has the most published articles, followed by China. Several European countries follow; and if taken as a whole, Europe has produced more studies than the USA. Only three studies have been published from African countries. These trends suggest that QOL research is being conducted mainly in developed countries. A Chinese review of QOL studies from 2009 commented that such studies in China were rare and that the research was conducted predominantly in the West [9]. Shek [9] argued that this can be explained by the socioeconomic and political circumstances, in addition to cultural differences, such as different sets of values and philosophical foundations. It is possible that the concept of QOL is understood differently in different cultures, and the relevance from the cross-cultural context is unclear. Therefore, it is of interest to conduct more QOL studies in Asian and other non-Western cultures to understand QOL and its manifestation from the cross-cultural context. Our snapshot review suggests that the situation is changing and that QOL research is expanding in China.

The studies included in our review show that QOL research has involved primarily patient groups with specified diseases, especially different kinds of cancer and other long-term diseases. Improved medical treatment means that more people are living with disease and chronic conditions. This has led to an increasing interest in QOL research by focusing not only on treatment options and effect, but also on the effects on people’s lives. Fewer studies have focused on community samples and children. Only 12% of the included studies involved children or adolescents. There are several possible explanations for the focus on adults, primarily that the prevalence of disease and long-term conditions is much lower in children than in adults. There are also challenges in the assessment of QOL in children and adolescents, including conceptual, methodological, and practical aspects. Ravens-Sieberer et al. [10] identified issues such as the relevance and age-appropriate tools to measure QOL in children, challenges in using proxy-rated QOL measures in children, and cross-cultural comparison of the dimensions of QOL.

The research designs of the included studies included descriptive, longitudinal, and experimental designs. QOL is increasingly used as an endpoint in clinical trials, often as part of an evaluation of different treatment or intervention outcomes. It is noteworthy that many of the interventions described in the included studies are not intended to increase QOL and therefore, QOL appears as an important, but secondary, outcome. Including QOL as a secondary outcome emphasizes the importance of such issues when assessing the benefits of different treatment options; that is, researchers are interested in both the medical outcomes as well as the effects of treatment on patients’ lives. This can provide information to clinicians and policymakers about how best to prioritize and allocate resources within health care.

One of the critiques of QOL research is the lack of conceptual clarity and a uniform definition of QOL [6]. Using a clearer and definitive definition of QOL research and research that includes QOL measures may increase the conceptual understanding, which will help researchers plan and conduct more rigorous QOL research studies [6].

Only one study in the review had a mixed-methods design, and only one was purely qualitative. Mixed methods involve the collection and analysis of both quantitative and qualitative data [11]. Traditionally, QOL research has been quantitative and there are few qualitative studies, although during the past years, an increasing number of qualitative QOL studies have added an important dimension to QOL research [12]. However, because of the few qualitative studies and the limited search (1 week), we have not been able to identify whether the number of qualitative studies has increased in recent years.

QOL measures can be categorized into three subtypes according to the type of report (self-report vs. proxy report), scores (single indicator, profile, or battery approach), and population (generic vs. condition specific), which allows for classification based on the scope and applicability of the study [13]. This review found that a diverse number of different measures are used to evaluate QOL. Most of the studies included a condition-specific measure, which is not surprising given that various disease populations were the target groups in most of the included studies. Generic measures of QOL are used either alone or in combination with a condition-specific instrument. Using both generic and condition-specific instruments has an advantage, because generic instruments can be used to compare QOL between health conditions, and condition-specific measures specifically address the health condition and appear to be more clinically relevant [14]. The choice of the type of measure clearly depends on the aim(s) of the study. The findings of our review indicate that a measure seems to exist for every disease. The challenge is to find instruments that can be widely used but have good psychometric properties for every health condition. The generic measures used in the included studies are well known and widely used and have been well validated across cultures. Examples are the SF-36, EQ-5D, and WHOQOL-BREF for adults, and Kidscreen, CHQ, and PedsQL for children.

QOL research has been criticized for a lack of conceptual clarity and clear definition of QOL [8, 15–17]. In this snapshot review, most articles had a low-quality score according to the criteria of Gill and Feinstein [5, 8]. Surprisingly, only 13% of the articles provided a definition of the concept of QOL. This is lower than that reported in the survey of Bratt and Moons [7], which found that 27% of the studies of congenital health disease from 2005 to 2014 provided a definition of QOL. A definition of QOL should state clearly what the authors mean by QOL and how it is related to other concepts [18]. The criteria fulfilled most frequently in our study were stating the domains of QOL to be measured, giving a reason for choosing the instruments used, and aggregating the results from multiple items. This is consistent with the results of Bratt and Moons [7]. It is important to give the reason for choosing an instrument. Valid measurements methods require that the instruments employed are suitable for the intended task [7]. Our results showed that in 25% of the studies, the authors gave reasons for choosing an instrument. For instance, pointed Hubert-Dibon et al. [17] out that they chose the KIDSCREEN-27questionnaire because the instrument provides a broad perspective on understanding of HRQOL, it includes five dimensions and requires only 10–15 min to complete, but still permits evaluation of the main components of HRQOL [17]. However, few studies have distinguished QOL from HRQOL, only 6% of the articles found in our study did so. According to Moons et al. [19], it is important to report and state clearly whether overall QOL or HRQOL has been measured. The majority of the included studies measured HRQOL, and only few articles distinguished between the terms. Cuerda et al. [20] argued for instance that they preferred to study HRQOL because it is a dynamic variable, which evaluates the subjective influence of health status, health care, and preventive health activities [20]. The terms health, HRQOL, and QOL are often used interchangeably in the literature. However, these terms have different definitions and intended use, and it is problematic that some researchers fail to distinguish between them. Further, it is debated whether many of the instruments used to measure HRQOL actually measure self-perceived health status and that the term (HR)QOL is unjustified [21].

Based on our evaluation of methodological and conceptual clarity, we conclude that most QOL studies in health and medicine have conceptual and methodological limitations. In general, theories and theoretical frameworks improve the understanding of QOL. The use of theoretical perspectives in empirical research deepens understanding and can help to establish new knowledge about QOL [22]. Theory is a presupposition for the ability to compare results from different studies and is important in the development and testing of QOL measures. Basing research on theory also improves the conceptual clarity and therefore the validity of the measures. The application of theoretical thinking leads to hypothesis generation, which makes research cumulative instead of atomistic. However, theoretical thinking needs to be interwoven in all stages of research. Its absence might engender a static concept of QOL by continuing to test the same parameters. Both qualitative and theoretical approaches to QOL are needed to open up the concept for discussion and change.

## Strengths and limitations

One strength of this snapshot is that we searched widely in databases: Scopus, which covers Embase and MEDLINE, CINAHL, and PsycINFO. Another strength is that the selection process and review were performed independently by pairs of researchers and that agreement was reached in a consensus meeting.

However, the present study has some limitations. First, this study was designed as a snapshot and aimed to analyze and describe QOL research in one random week. Admittedly, a snapshot of a single week might not be representative of QOL research in general. However, a large number of QOL studies are published every year. A random selection can give a good picture of QOL research. To ensure that this week was not unique in terms of the number of articles published, we performed the same search strategy of the same databases for one random week 2 months later, and this search yielded nearly the same number of articles and showed the same trends in the type of articles, countries of origin, and study design. Second, searches were limited to English language only. It is possible that similar studies may have been published in other languages than English.

Third, the criteria used were developed in 1994, and one may question whether these remain relevant in 2018. However, the criteria were refined by Moons in 2004 and, to our knowledge, no other criteria for assessing the conceptual rigor in QOL studies have been published.

## Conclusion

Knowledge about QOL is important for understanding the consequences of illness and treatment, and for medical decision-making across age groups and culture. QOL is an important endpoint in medical and health research, and QOL research involves a variety of target groups and research designs. However, based on the current evaluation of the methodological and conceptual clarity of QOL research, we conclude that many QOL studies in health and medicine have conceptual and methodological challenges. There is a need for improvements in this field, and researchers should pay closer attention to methodological and conceptual issues when planning QOL studies.

## Electronic supplementary material

Below is the link to the electronic supplementary material.

Electronic supplementary material 1 (DOCX 59 kb)
