# Supplementary material for: A systematic review of quality of life research in medicine and health sciences
Source: Qual Life Res. 2019 Jun 11;28(10):2641–50. doi: 10.1007/s11136-019-02214-9 (PMC6761255; doi:10.1007/s11136-019-02214-9)
Supplement: Supplementary file 1 — Electronic supplementary material 1 (DOCX 59 kb) [file 11136_2019_2214_MOESM1_ESM.docx]

Appendix; Reference list of reviewed papers

A systematic review of quality of life research in medicine and health sciences-", QURE-D-18-01301R1.

1. Alfian S. D., H. Sukandar, K. Lestari and R. Abdulah (2016). Medication Adherence Contributes to an Improved Quality of Life in Type 2 Diabetes Mellitus Patients: A Cross-Sectional Study, *Diabetes Ther* (2016) 7: 755.
2. Anu M., K. Suresh and P. L. Basavanna (2016) .A cross-sectional study of quality of life among subjects with epilepsy attending a tertiary care hospital*, J Clin Diagn Re*s.; 10(12): OC13–OC15.
3. Aravena P. C., M. Brandt, F. Klett, M. Hernández and C. Coronado (2016).Effect of presurgical orthopedics on oral-health related quality of life in Chilean children with cleft lip and palate. A pilot study, J Oral Res (2016); 5(7): 266-270.
4. Armstrong A. W., D. G. Villanueva Quintero, C. M. Echeverría, Y. Gu, M. Karunaratne and O. Reyes Servín (2016) Body Region Involvement and Quality of Life in Psoriasis: Analysis of a Randomized Controlled Trial of Adalimumab*, Am J Clin Dermatol*. 17(6):691-699.
5. Asnaani A., A. N. Kaczkurkin, E. Alpert, C. P. McLean, H. B. Simpson and E. B. Foa. (2016) The effect of treatment on quality of life and functioning in OCD, *Compr Psychiatry.* b; 73:7-14.
6. Ayres E. L., A. Costa, A. C. F. Jorge, J. E. G. Júnior, M. Szrajbman and B. Sant'Anna (2016) "Monocentric prospective study for assessing the efficacy and tolerability of a cosmeceutical formulation in patients with melasma, *Surgical and Cosmetic Dermatology* 8(3)
7. Banno T., T. Hasegawa, Y. Yamato, S. Kobayashi, D. Togawa, S. Oe, Y. Mihara and Y. Matsuyam (2016) T1 pelvic angle is a useful parameter for postoperative evaluation in adult spinal deformity patients*, SPINE*. 41(21):1641–1648, NOV (2016)
8. Bao C., P. Liu, H. Liu, X. Jin, V. D. Calhoun, L. Wu, Y. Shi, J. Zhang, X. Zeng, L. Ma, W. Qin, J. Zhang, X. Liu, J. Tian and H. Wu (2016) Different brain responses to electro-acupuncture and moxibustion treatment in patients with Crohn's disease, *Scientific Reports* volume 6, Article number: 36636 (2016)
9. Baumert B. G., M. E. Hegi, M. J. van den Bent, A. von Deimling, T. Gorlia, K. Hoang-Xuan, Nordal, J. Rees, D. Lacombe, W. P. Mason and R. Stupp (2016) Temozolomide chemotherapy versus radiotherapy in high-risk low-grade glioma (EORTC 22033-26033): a randomised, open-label, phase 3 intergroup study*, Lancet Oncol*. ;17(11):1521-1532.
10. Benavent J. V., C. Igual, E. Mora, R. Antonio and J. M. Tenias (2016). Cross-cultural validation of the Prosthesis Evaluation Questionnaire in vascular amputees fitted with prostheses in Spain, *Prosthet Orthot Int.* ;40(6):713-719
11. Berman J., A. Aran, T. Berenstein-Weyel and E. Lebel (2016). Exploring the association between Legg-Calvé-Perthes disease and attention deficit hyperactivity disorder in children*, Isr Med Assoc* ;18(11):652-654.
12. Boisen S., C. Krägeloh, D. Shepherd, C. Ryan, J. Masters, S. Osborne, R. D. MacLeod, M. Gray and J. W. Keogh (2016).A cross-sectional comparison of quality of life between physically active and underactive older men with prostate cancer, *J Aging Phys Act*. ;24(4):642-648.
13. Boonpiyathad T., P. Pradubpongsa and A. Sangasapaviriya (2016) Vitamin d supplements imquality of life in chronic spontaneous urticaria patients: A prospective case-control study*, Dermatoendocrinol. 16;6: e29727*.
14. Bosia M., M. Buonocore, M. Bechi, M. Spangaro, A. Pigoni, M. Croci, F. Cocchi, C. Guglielmino, L. Bianchi, E. Smeraldi and R. Cavallaro (2016). Cognitive remediation and functional improvement inschizophrenia: Is it a matter of size*? Eur Psychiatry*.;40:26-32.
15. Bouman M. B., W. B. van der Sluis, L. E. van Woudenberg Hamstra, M. E. Buncamper, B. P. C. Kreukels, W. J. H. J. Meijerink and M. G. Mullender (2016). Patient-Reported Esthetic and Functional Outcomes al Hypoplasia*, J Sex Med*.;13(9):1438-1444.
16. Burns T. M., R. Sadjadi, K. Utsugisawa, K. G. Gwathmey, A. Joshi, S. Jones, V. Bril, C. Barnett, J. T. Guptill, D. B. Sanders, L. Hobson-Webb, V. C. Juel, J. Massey, K. L. Gable, N. J. Silvestri, G. Wolfe, G. Cutter, Y. Nagane, H. Murai, M. Masuda, M. E. Farrugia, C. Carmichael, S. Birnbaum, J. Y. Hogrel, S. Nafissi, F. Fatehi, C. Ou, W. Liu and M. Conaway (2016). International clinimetric evaluation of the MG-QOL15, resulting in slight revision and subsequent validation of the MG-QOL15r*, Muscle Nerve.*;54(6):1015-1022.
17. Burns-Lynch B., E. Brusilovskiy and M. S. Salzer (2016). An empirical study of the relationship between community participation, recovery, and quality of life of individuals with serious mental illnesses*, Isr J Psychiatry Relat Sci*. ;53(1):46-54
18. Büyükkaragöz B., N. Buyan, N. Doğrucan and B. Çelik (2016).Health-related quality of life of pediatric renal transplant recipients and their parents: The role of associated factors and clinical counseling, *Turk J Med Sci.* 17;46(5):1481-1485.
19. Camerota F., C. Celletti, E. Di Sipio, C. De Fino, C. Simbolotti, M. Germanotta, M. Mirabella, L. Padua and V. Nociti (2016). Focal muscle vibration, an effective rehabilitative approach in severe gait impairment due to multiple sclerosis, *J Neurol* Sci 15;372:33-39.
20. [Cao TT](https://www.ncbi.nlm.nih.gov/pubmed/?term=Cao%20TT%5BAuthor%5D&cauthor=true&cauthor_uid=27779168), [Sun XL](https://www.ncbi.nlm.nih.gov/pubmed/?term=Sun%20XL%5BAuthor%5D&cauthor=true&cauthor_uid=27779168), [Wang SY](https://www.ncbi.nlm.nih.gov/pubmed/?term=Wang%20SY%5BAuthor%5D&cauthor=true&cauthor_uid=27779168) [Yang X](https://www.ncbi.nlm.nih.gov/pubmed/?term=Yang%20X%5BAuthor%5D&cauthor=true&cauthor_uid=27779168) [Wang JL](https://www.ncbi.nlm.nih.gov/pubmed/?term=Wang%20JL%5BAuthor%5D&cauthor=true&cauthor_uid=27779168) (2016) Porcine Small Intestinal Submucosa Mesh for Treatment of Pelvic Organ Prolapsed. [*Chin Med J (Engl).*](https://www.ncbi.nlm.nih.gov/pubmed/27779168)*5;*129(21):2603-2609.
21. Cannon S., K. Lawry, M. Brudell, R. Rees, R. Wenke and L. Bisset (2016) Appetite for change: a multidisciplinary team approach to behavioral modification for weight management in a community health group setting, *Eat Weight Disord*. ;21(4):661-668.
22. Casellas F., D. Ginard and S. Riestra (2016). Patient satisfaction in the management of mild-to-moderate ulcerative colitis: Results of a Delphi study among patients and physicians, *Dig Liver Dis*. ;48(10):1172-9.
23. Chang C. Y., L. J. H. Lee, J. D. Wang, C. T. Lee, C. M. Tai, T. Q. Tang and J. T. Lin (2016). Health-related quality of life in patients with Barrett's esophagus, *Health Qual Life Outcomes.* 14;14(1):158.
24. Cheon J. J., J. Y. Uhm, G. H. Kang, E. G. Kang, S. Y. Kim and S. S. Chang (2016). Evaluation of the dermatologic life quality among cleanroom workers in a secondary battery factory*, Ann Occup Environ Med*. 2;28(1):39.
25. Choi D. K., H. B. Jung, Y. G. Lee, K. K. Kim and S. T. Cho (2016). A sequential comparison of postoperative voiding function between two different transobturator sling procedures, *Can Urol Assoc J.* 10(11-12): E372-E376.
26. Ciorogar G., F. Zaharie, A. Ciorogar, D. Birta, A. Degan, I. Balint, E. Mois, F. Graur and C. Iancu, HVM Bioflux (2016) Quality of life outcomes in patients living with stoma, *HVM Bioflux;8(3):137-140.*
27. Cofield S. S., R. J. Fox, T. Tyry, A. R. Salter and D. Campagnolo (2016). Disability progression after switching from natalizumab to fingolimod or interferon beta/glatiramer acetate therapies: A NARCOMS analysis, *Int J MS Care18(5):230-238.*
28. Coleman C. L.(2017). Health related quality of life and depressive symptoms among seropositive African Americans, *Appl Nurs Res. ;*33:138-141.
29. Cuerda M. C., A. Apezetxea, L. Carrillo, F. Casanueva, F. Cuesta, J. A. Irles, M. N. Virgili, M. Layola and L. Lizan (2016). Development and validation of a specific questionnaire to assess health-related quality of life in patients with home enteral nutrition: NutriQoL® development, *Patient Prefer Adherence*. 4; 10:2289-2296
30. de Boer S. M., M. E. Powell, L. Mileshkin, D. Katsaros, P. Bessette, C. Haie-Meder, P. B. Ottevanger, J. A. Ledermann, P. Khaw, A. Colombo, A. Fyles, M. H. Baron, H. C. Kitchener, H. W. Nijman, R. F. Kruitwagen, R. A. Nout, K. W. Verhoeven-Adema, V. T. Smit, H. Putter and C. L. Creutzberg (2016).Toxicity and quality of life after adjuvant chemoradiotherapy versus radiotherapy alone for women with high-risk endometrial cancer (PORTEC-3): an open-label, multicentre, randomised, phase 3 trial, *Lancet Oncol*.;17(8):1114-1126.
31. De Graaff A. A., J. Van Lankveld, L. J. Smits, J. J. Van Beek and G. A. J. Dunselman (2016). Dyspareunia and depressive symptoms are associated with impaired sexual functioning in women with endometriosis, whereas sexual functioning in their male partners is not affected, *Hum Reprod.*;31(11):2577-2586
32. de Miranda A. L. C., L. L. D. Szerwieski, M. D. Ferreira, M. C. de Miranda and L. E. R. Cortez (2016).Perception and quality of life of patients after surgery keratoconus, *Rev.* *bras.oftalmol*. vol.75 no.5
33. De Souza A. C. C., T. M. M. Moreira, E. S. De Oliveira, A. V. B. De Menezes, A. M. O. Loureiro, C. B. De Araújo Silva, J. G. Linard, I. L. S. De Almeida, S. M. Mattos and J. W. P. Borges (2016). Effectiveness of educational technology in promoting quality of life and treatment adherence in hypertensive people, *PLoS One*. 16;11(11):
34. Diegelmann M., O. K. Schilling and H. W. Wahl (2016).Feeling blue at the end of life: Trajectories of depressive symptoms from a distance-to-death perspective, *Psychol Aging.*;31(7):672-686
35. Ding W., S. Chen, R. Wang, J. Cai, Y. Cheng, L. Yu, Q. Li, F. Deng, S. Zhu and W. Yu (2016). Percutaneous radiofrequency thermocoagulation for trigeminal neuralgia using neuronavigationguided puncture from a mandibular angle, *Medicine (Baltimore*).95(40): e4940.
36. Ditzler N. and M. Greenhawt (2016). Influence of health literacy and trust in online information on food allergy quality of life and self-efficacy (2016)*, Ann Allergy Asthma Immunol.* 117(3):258-263.e1.
37. Dong A., X. Chen, L. Zhu, L. Shi, Y. Cai, B. Shi, L. Shao and W. Guo (2016). Translation and validation of a Chinese version of the Warwick–Edinburgh Mental Well-being Scale with undergraduate nursing trainees, *J Psychiatr Ment Health Nurs.* ;23(9-10):554-560.
38. Doorenbos A. Z., W. C. Levy, J. R. Curtis and C. M. Dougherty (2016). An Intervention to Enhance Goals-of-Care Communication Between Heart Failure Patients and Heart Failure Providers, *J Pain Symptom Manage. ;*52(3):353-60.
39. Eliacik K., N. Bolat, C. Koçyiğit, A. Kanik, E. Selkie, H. Yilmaz, G. Catli, N. O. Dundar and B. N. Dundar (2016).Internet addiction, sleep and health-related life quality among obese individuals: a comparison study of the growing problems in adolescent health*, Eat Weight Disord*. ;21(4):709-717.
40. Fatiregun O. A., A. T. Olagunju, A. R. Erinfolami, O. A. Arogunmati, O. A. Fatiregun and J. D. Adeyemi (2017). Relationship between anxiety disorders and domains of health-related quality of life among Nigerians with breast cancer, *The Breast.*; 31:150-156.
41. Feldman S. R., D. Thaçi, M. Gooderham, M. Augustin, C. de la Cruz, L. Mallbris, M. Buonanno, S. Tatulych, M. Kaur, S. Lan, H. Valdez and C. Mamolo (2016). Tofacitinib improves pruritus and health-related quality of life up to 52 weeks: Results from 2 randomized phase III trials in patients with moderate to severe plaque psoriasis, J Am *Acad Dermatol*. (2016) ;75(6):1162-1170.e3
42. Feng J., X. Wang, X. Li, D. Zhao and J. Xu (2016)."Acupuncture for chronic obstructive pulmonary disease (COPD): A multicenter, randomized, sham-controlled trial, *Medicine* 95(40): e4879,
43. Fischer K., P. de Kleijn, C. Negrier, E. P. Mauser-Bunschoten, P. R. van der Valk, K. P. M. van Galen, A. Willemze and R. Schutgens (2016). The association of haemophilic arthropathy with Health-Related Quality of Life: a post hoc analysis, *Haemophilia.* ;22(6):833-840
44. Franke K. J., U. Domanski, M. Schroeder, V. Jansen, F. Artmann, U. Weber, R. Ettler and G. Nilius (2016).Telemonitoring of home exercise cycle training in patients with COPD, Int J *Chron Obstruct Pulmon Dis*. 11; 11:2821-2829.
45. Furtado R. V., S. J. Vivian, H. Van Der Wall and G. L. Falk (2016) Medium-term durability of giant hiatus hernia repair without mesh*, Ann R Coll Surg Engl*. ;98(7):450-5.
46. Galiano-Castillo N., M. Arroyo-Morales, A. Ariza-Garcia, C. Sánchez-Salado, C. Fernández-Lao, I. Cantarero-Villanueva and L. Martín-Martín (2016) . The six-minute walk test as a measure of health in breast cancer patients*, J Aging Phys Act*.;24(4):508-515
47. Gerber M., K. Endes, S. Brand, C. Herrmann, F. Colledge, L. Donath, O. Faude, U. Pühse, H. Hanssen and L. Zahner (2016).In 6- to 8-year-old children, hair cortisol is associated with body mass index and somatic complaints, but not with stress, health-related quality of life, blood pressure, retinal vessel diameters, and cardiorespiratory fitness, *Psychoneuroendocrinology.* 76:1-10.
48. Gill S., Y. J. Ko, C. Cripps, A. Beaudoin, S. Dhesy-Thind, M. Zulfiqar, P. Zalewski, T. Do, P. Cano, W. Y. H. Lam, S. Dowden, H. Grassin, J. Stewart and M. Moore (2016).PANCREOX: A randomized phase III study of fluorouracil/leucovorin with or without oxaliplatin for second-line advanced pancreatic cancer in patients who have received gemcitabine-based chemotherapy*, J Clin Oncol. 10;34(32):3914-3920.*
49. Guan L., Y. Xiang, X. Ma, Y. Weng and W. Liang (2016). Qualities of life of patients with psychotic disorders and their family caregivers: Comparison between hospitalised and community-based treatment in Beijing, China, *PLoS One. 21;11(11): e0166811*
50. Guo J. G., Y. Fei, B. Huang and M. Yao (2016). CT-guided thoracic sympathetic blockade for palmar hyperhidrosis: Immediate results and postoperative quality of life, *J Clin Neurosci.;* 34:89-93.
51. Gutiérrez-Sánchez D., J. P. Leiva-Santos, R. Sánchez-Hernández, D. Hernández-Marrero and A. I. Cuesta-Vargas (2016). Spanish modified version of the palliative care outcome scale-symptoms renal: cross-cultural adaptation and validation, *BMC Nephrol.* 18;17(1):180.
52. Hendred S. K. and E. R. Foster (2016). Use of the World Health Organization Quality of Life Assessment Short Version in Mild to Moderate Parkinson Disease, *Arch Phys Med Rehabil*., 97(12):2123-2129.
53. Hisam A., F. Ashraf, M. N. Rana, Y. Waqar, S. Karim and F. Irfan (2016) Health related quality of life in patients with single lower limb amputation, *J Coll Physicians Surg* Pak. ;26(10):851-854
54. Hochman M. J., A. H. Kamal, S. P. Wolf, G. P. Samsa, D. C. Currow, A. P. Abernethy and T. W. LeBlanc (2016).Anticholinergic Drug Burden in Noncancer Versus Cancer Patients Near the End of Life*, J Pain Symptom Manage*;52(5):737-743.
55. Holbrook J. T., E. A. Sugar, R. H. Brown, L. T. Drye, C. G. Irvin, A. R. Schwartz, R. S. Tepper, R. A. Wise, R. Z. Yasin and M. F. Busk (2016). Effect of continuous positive airway pressure on airway reactivity in asthma: A randomized, sham-controlled clinical trial, *Ann Am Thorac* Soc.;13(11):1940-1950.
56. Howells P., D. Thickett, C. Knox, D. Park, F. Gao, O. Tucker, T. Whitehouse, D. McAuley and G. Perkins (2016). The impact of the acute respiratory distress syndrome on outcome after esophagectomy, *Br J Anaesth*.;117(3):375-81.
57. Hsueh Y. M., W. J. Chen, C. Y. Lee, S. N. Chien, H. S. Shiue, S. R. Huang, M. I. Lin, S. C. Mu and R. L. Hsieh (2016). Association of Arsenic Methylation Capacity with Developmental Delays and Health Status in Children: A Prospective Case-Control Trial, *Sci Rep. 17; 6:37287.*
58. Huang W., Z. Huang, G. Xiao and X. Qin (2016).Effect of transurethral split of the

prostate using a double-columnar balloon catheter for benign prostatic hyperplasia: A single-center experience of 565 consecutive patients, *Medicine;*95(40): e4657.

1. Hubert-Dibon G., M. Bru, C. G. Le Guen, E. Launay and A. Roy (2016). Health-related quality of life for children and adolescents with specific language impairment: A cohort study by a Learning Disabilities Reference Center, *PLoS One.* 16;11(11):0166541.
2. Hugele F., L. Panel, C. Farache, A. Kashef, A. Cornille and C. Courtieu (2017). Two years follow up of 270 patients treated by transvaginal mesh for anterior and/or apical prolapse*,Eur J Obstet Gynecol Reprod Biol.* ;208:16-22.
3. Hynes D. M., M. J. Fischer, L. A. Schiffer, R. Gallardo, I. B. Chukwudozie, A. Porter, M. Berbaum, J. Earheart and M. L. Fitzgibbon (2016). Evaluating a novel health system intervention for chronic kidney disease care using the RE-AIM framework: Insights after two years*, Contemp Clin Trials*;52:20-26.
4. Jácome C. and A. Marques (2016). Short- and long-term effects of pulmonary rehabilitation in patients with mild COPD: A comparison with patients with moderate to severe COPD, *J Cardiopulm Rehabil Prev*;36(6):445-453.
5. Jakel P., J. Kenney, N. Ludan, P. S. Miller, N. McNair and E. Matesic (2016).Effects of the use of the Provider Resilience mobile application in reducing compassion fatigue in oncology nursing, *Clin J Oncol Nurs*. 1;20(6):611-616.
6. Johnson T. M., II, C. P. Vaughan, P. S. Goode, D. L. Bliwise, A. D. Markland, C. Huisingh, D. T. Redden, G. McGwin, Jr., R. Eisenstein, J. G. Ouslander, M. Issa and K. L. Burgio (2016). Pilot Results from a Randomized Trial in Men Comparing Alpha-Adrenergic Antagonist versus Behavior and Exercise for Nocturia and Sleep, *Clin Ther.* 28. pii: S0149-2918(16)30742-1
7. Johnston S. L., M. Szigeti, M. Cross, C. Brightling, R. Chaudhuri, T. Harrison, A. Mansur, L. Robison, Z. Sattar, D. Jackson, P. Mallia, E. Wong, C. Corrigan, B. Higgins, P. Ind, D. Singh, N. C. Thomson, D. Ashby and A. Chauhan (2016).Azithromycin for acute exacerbations of asthma: The AZALEA randomized clinical trial, *JAMA Intern Med.* 1;176(11):1630-1637.
8. Khoramzadeh S., N. Saki, I. Davoodi, M. Nosratabadi and A. Yadollahpour (2016). Investigating the Therapeutic Efficacy of Neurofeedback Treatment on the Severity of Symptoms and Quality of Life in Patients with Tinnitus, *Int J Ment Health Addiction* 14: 982.
9. Kim S. H., S. I. Seo, H. M. Lee, H. Y. Choi, S. H. Jeon, H. L. Lee, T. G. Kwon, Y. J. Kim, W. J. Kim and J. Chung (2016). A prospective multicenter trial of the efficacy and tolerability of neoadjuvant sunitinib for inoperable metastatic renal cell carcinoma*, J Korean Med Sci.* ;31(12):1983-1988.
10. Kimball A. B., T. Luger, A. Gottlieb, L. Puig, R. Kaufmann, E. Nikaï, B. Zhu, E. Edson-Heredia, H. Carlier, C. Y. Lin, O. Goldblum and G. Yosipovitch (2016). Impact of ixekizumab on psoriasis itch severity and other psoriasis symptoms: Results from 3 phase III psoriasis clinical trials*, J Am Acad Dermatol.* 75(6):1156-1161.
11. Kirkøen B., P. Berstad, E. Botteri, L. Bernklev, B. El-Safadi, G. Hoff, T. De Lange and T. Bernklev (2016).Psychological effects of colorectal cancer screening: Participants vs individuals not invited*, World J Gastroenterol*. 21;22(43):9631-9641.
12. Klassen A. F., S. J. Cano, A. Alderman, C. East, L. Badia, S. B. Baker, S. Robson and A. L. Pusic (2016). Self-report scales to measure expectations and appearance-related psychosocial distress in patients seeking cosmetic treatments, *Aesthet Surg* J. 36(9):1068-78.
13. Kolluri R., K. Gibson, D. Cher, M. Madsen, R. Weiss and N. Morrison (2016). Roll-in phase analysis of clinical study of cyanoacrylate closure for incompetent great saphenous veins, J *Vasc Surg Venous Lymphat Disord*. (2016) Oct;4(4):407-15
14. Krawczyk-Ozóg A., T. Tokarek, K. Moczala, Z. Siudak, A. Dziewierz, W. Mielecki, T. Górecki, K. Gerba and D. Dudek (2016). Long-term quality of life and clinical outcomes in patients with resistant hypertension treated with renal denervation, *Postepy Kardiol Interwencyjnej.* (2016);12(4):329-333
15. Krebber A. M. H., F. Jansen, B. I. Witte, P. Cuijpers, R. de Bree, A. Becker-Commissaris, E. F. Smit, A. van Straten, A. M. Eeckhout, A. T. F. Beekman, C. R. Leemans and I. M. Verdonck-de Leeuw (2016). Stepped care targeting psychological distress in head and neck cancer and lung cancer patients: A randomized, controlled trial, *Ann Oncol* 27(9):1754-60.
16. Krističević T., M. Hrženjak and G. Sporiš (2016). Effects of 24-week aerobic training protocol on physiological abilities and quality of life of free-living elderly females, *Sport Science 9*  Suppl 2: 98-103 X
17. Krüger J., P. J. Meffert, L. J. Vogt, S. Gärtner, A. Steveling, M. Kraft, J. Mayerle, M. M. Lerch and A. A. Aghdassi (2016). Early parenteral nutrition in patients with biliopancreatic mass lesions, a prospective, randomized intervention trial, *PLoS One.* 18;11(11).
18. Kuck K. H., A. Fürnkranz, K. R. J. Chun, A. Metzner, F. Ouyang, M. Schlüter, A. Elvan, H. W. Lim, F. J. Kueffer, T. Arentz, J. P. Albenque, C. Tondo, M. Kühne, C. Sticherling and J. Brugada (2016). Cryoballoon or radiofrequency ablation for symptomatic paroxysmal atrial fibrillation: Reintervention, rehospitalization, and quality-of-life outcomes in the FIRE and ICE trial, *Eur Heart J*. Oct 7;37(38):2858-2865
19. Kularatna S., J. Byrnes, Y. K. Chan, M. J. Carrington, S. Stewart and P. A. Scuffham (2016) Comparison of contemporaneous responses for EQ-5D-3L and Minnesota Living with Heart Failure a case for disease specific multiattribute utility instrument in cardiovascular conditions, *Int J Cardiol.* 15; 227:172-176
20. Kyrklund K., S. Taskinen, R. J. Rintala and M. P. Pakarinen (2016). Sexual Function, Fertility and Quality of Life after Modern Treatment of Anorectal Malformations*, J Urol.* ;196(6):1741-1746.
21. Lauche R., J. Spitzer, B. Schwahn, T. Ostermann, K. Bernardy, H. Cramer, G. Dobos and J. Langhorst (2016). Efficacy of cupping therapy in patients with the fibromyalgia syndrome-a randomised placebo-controlled trial, *Sci Rep.* 17; 6:37316.
22. Lazzarotto S., K. Baumstarck, A. Loundou, Z. Hamidou, V. Aghababian, T. Lero and P. Auquier(2016). Age-related hearing loss in individuals and their caregivers: Effects of coping on the quality of life among the dyads, *Patient Prefer Adherence*. 7; 10:2279-2287
23. Lechtzin N., S. Allgood, G. Hong, K. Riekert, J. A. Haythornthwaite, P. Mogayzel, J. Hankinson and M. Yaster (2016). The Association Between Pain and Clinical Outcomes in Adolescents With Cystic Fibrosis, *J Pain Symptom Manage*;52(5):681-687.
24. Lee L. K., N. Ebata, P. Hlavacek, M. DiBonaventura, J. C. Cappelleri and A. Sadosky (2016) Humanistic and economic burden of fibromyalgia in Japan*, J Pain Res*. 4;9:967-978
25. Lei J. Y., L. N. Yan, W. T. Wang, J. Q. Zhu and D. J. Li (2016). Health-Related Quality of Life and Psychological Distress in Patients With Early-Stage Hepatocellular Carcinoma After Hepatic Resection or Transplantation*, Transplant Proc*.;48(6):2107-11.
26. Li Y., L. Stocchi, X. Mu, D. Cherla and F. H. Remzi (2016) Long-term Outcomes of Sphincter-Saving Procedures for Diffuse Crohn's Disease of the Large Bowel, *Dis Colon Rectum;59(12):1183-1190*.
27. Li Y., W. B. Zhao, D. L. Wang, Q. He, Q. Li, F. X. Pei and L. Liu (2016). Treatment of osteoporotic intertrochanteric fractures by zoledronic acid injection combined with proximal femoral nail anti-rotation*, Chin J Traumatol*. 1;19(5):259
28. Liao C. H. and H. C. Kuo (2016). High satisfaction with direct switching from antimuscarinics to mirabegron in patients receiving stable antimuscarinic treatment*, Medicine (Baltimore).* 201;95(45): e4962.
29. Lindhardt T. and M. H. Nielsen (2017). Older patients’ use of technology for a post-discharge nutritional intervention – A mixed-methods study, *Int J Med Inform*; 97:312-321.
30. Lock J., H. Raat, M. Peters, M. Scholten, M. Beijlevelt, R. Oostenbrink, F. W. G. Leebeek, H. A. Moll and M. H. Cnossen (2016). Optimization of home treatment in haemophilia: effects of transmural support by a haemophilia nurse on adherence and quality of life, *Haemophilia. 22*(6):841-851.
31. Loiselle K. A., R. R. Ramsey, J. R. Rausch and A. C. Modi (2016). Trajectories of Health-Related Quality of Life among Children with Newly Diagnosed Epilepsy, *J Pediatr Psychol*. ;41(9):1011-21
32. Lomper K., Chudiak A, Uchmanowicz I, Rosinczukj janowska-polanska B (2016) Effects of depression ands anxiety on asthma-related quality of life. *Pneumonol Alerg Pol.* 84(4):212-21
33. Lopez-Villegas A., D. Catalan-Matamoros, E. Robles-Musso and S. Peiro (2016). Effectiveness of pacemaker tele-monitoring on quality of life, functional capacity, event detection and workload: The PONIENTE trial, *Geriatr Gerontol Int*. ;16(11):1188-1195.
34. Maroufizadeh S., A. Ghaheri, P. Amini and R. Omani Samani (2017).Psychometric properties of the fertility quality of life instrument in infertile Iranian women, *Int J Fertil Steril; 10(4): 371–379*
35. Maheswari P Somasundram (2016). Health-related quality of life measurement in asthma and chronic obstructive pulmonary disease. [*Research Journal of Pharmacy and Technology*](https://www.scopus.com/sourceid/21100197160?origin=recordpage)Volume 9, Issue 5, Pages 518-520
36. Mengoni S. E., B. Gates, G. Parkes, D. Wellsted, G. Barton, H. Ring, M. E. Khoo, D. Monji-Patel, K. Friedli, A. Zia, L. Irvine and M. A. Durand (2016).Wordless intervention for people with epilepsy and learning disabilities (WIELD): A randomised controlled feasibility trial*, BMJ Open.* 10;6(11): 012993.
37. Michaud T. L., J. A. Nyman, E. Jutkowitz, D. Su, B. Dowd and J. M. Abraham (2016) Effect of workplace weight management on health care expenditures and quality of life, *J Occup Environ Med.* ;58(11):1073-1078.
38. Montirosso R., L. Giusti, A. Del Prete, R. Zanini, R. Bellù and R. Borgatti (2016)Does quality of developmental care in NICUs affect health-related quality of life in 5-y-old children born preterm*? Pediatr Res*; 80(6):824-828.
39. Mortazavi S. M. J., B. Haghpanah, M. M. Ebrahiminasab, T. Baghdadi and G. Toogeh (2016). Functional outcome of total knee arthroplasty in patients with haemophilia, *Haemophilia,* 22, 919–924
40. Muennig P. A., B. Mohit, J. Wu, H. Jia and Z. Rosen (2016). Cost Effectiveness of the Earned Income Tax Credit as a Health Policy Investment *Am J Prev* Med. ;51(6):874-881.
41. Nam S. H., H. J. Choi, W. D. Kang, S. M. Kim, M. C. Lim, S. Y. Park, J. S. Kim, B. G. Kim, D. S. Bae, J. W. Lee, T. J. Kim and T. Song (2016). Development and validation of the Korean version of hand-foot skin reaction and quality of life questionnaire (HF-QoL-K*), J Korean Med* Sci.;31(12):1969-1975
42. Ndikuno C, Namutebi J Kuteesa, Mukunya (2016). Quality of life of caregivers of patients diagnosed with severe mental illness at the national referral hospitals in Uganda*, BMC Psychiatry.* 15;16(1):400.
43. Neville C. E., J. Beneciuk, M. Bishop and M. Alappattu (2016). Analysis of physical therapy intervention outcomes for urinary incontinence in women older than 65 years in outpatient clinical settings, *Top Geriatr Rehabil*. ;32(4):251-25
44. Okkalides D. (2016) Thyroid Patient Salivary Radioiodine Transit and Dysfunction Assessment Using Chewing Gums, *Cancer Biother Radiopharm.;* 31(9):330-341.
45. Olthoff A., P. O. Carstens, S. Zhang, E. Von Fintel, T. Friede, J. Lotz, J. Frahm and J. Schmidt (2016). Evaluation of dysphagia by novel real-time *MRI, Neurology.* 15;87(20):2132-2138.
46. Paramita N., N. Nusdwinuringtyas, S. A. Nuhonni, T. D. Atmakusuma, R. I. Ismail, T. R. Mendoza and C. S. Cleeland (2016). Validity and Reliability of the Indonesian Version of the Brief Fatigue Inventory in Cancer Patients*, J Pain Symptom Manage*.; 52(5):744-751
47. Parikh K. S., A. Coles, P. J. Schulte, W. E. Kraus, J. L. Fleg, S. J. Keteyian, I. L. Piña, M. Fiuzat, D. J. Whellan, C. M. O'Connor and R. J. Mentz (2016). Relation of Angina Pectoris to Outcomes, Quality of Life, and Response to Exercise Training in Patients With Chronic Heart Failure (from HF-ACTION*), Am J Cardiol*. (2016) Ot 15;118(8):1211-1216
48. Park S. A., S. H. Chung and Y. Lee (2017).Factors influencing the quality of life of patients with advanced cancer, *Appl Nurs Res*.; 33:108-11
49. Pétré B., A. J. Scheen, O. Ziegler, A. F. Donneau, N. Dardenne, E. Husson, A. Albert and M. Guillaume (2016). Body image discrepancy and subjective norm as mediators and moderators of the relationship between body mass index and quality of life, *Patient Prefer Adherence.* 4; 10:2261-2270
50. Pilutti L. A., J. E. Paulseth, C. Dove, S. Jiang, M. P. Rathbone and A. L. Hicks (2016). Exercise training in progressive multiple sclerosis: A comparison of recumbent stepping and body weight-supported treadmill training*, Int J MS Care. ;18(*5):221-229.
51. Polat Ü., B. Bayrak Kahraman, İ. Kaynak and Ü. Görgülü (2016) Relationship among health-related quality of life, depression and awareness of home care services in elderly patients, *Geriatr Gerontol Int*. ;16(11):1211-1219.
52. Polizzotto M. N., T. S. Uldrick, K. M. Wyvill, K. Aleman, C. J. Peer, M. Bevans, I. Sereti, F. Maldarelli, D. Whitby, V. Marshall, P. H. Goncalves, V. Khetani, W. D. Figg, S. M. Steinberg, J. B. Zeldis and R. Yarchoan (2016). Pomalidomide for symptomatic Kaposi's sarcoma in people with and without HIV infection: A phase I/II study, *J Clin Oncol.* 34(34):4125-4131
53. Polley M. J., R. Jolliffe, E. Boxell, C. Zollman, S. Jackson and H. Seers (2016). Using a Whole Person Approach to Support People with Cancer: A Longitudinal, Methods Service Evaluation, *Integr Cancer Ther.* ;15(4):435-445
54. Prazeres F. and L. Santiago (2016). Relationship between health-related quality of life, perceived family support and unmet health needs in adult patients with multimorbidity attending primary care in Portugal: A multicentre cross-sectional study*, Health Qual Life Outcomes*. 11;14(1):156
55. Radsel A., D. Osredkar and D. Neubauer (2017). Health-related quality of life in children and adolescents with cerebral palsy, *Zdr Varst*. 28;56(1):1-10
56. Ran L., X. Jiang, E. Qian, H. Kong, X. Wang and Q. Liu (2016)."Quality of life, self-care knowledge access, and self-care needs in patients with colon stomas one-month post-surgery in a Chinese Tumor Hospital, *International J of Nursing Sciences* 3(3)
57. Rao A. D., E. A. Sugar, D. T. Chang, K. A. Goodman, A. Hacker-Prietz, L. M. Rosati, L. Columbo, E. O'Reilly, G. A. Fisher, L. Zheng, J. S. Pai, M. E. Griffith, D. A. Laheru, C. A. Iacobuzio-Donahue, C. L. Wolfgang, A. Koong and J. M. Herman (2016).Patient-reported outcomes of a multicenter phase 2 study investigating gemcitabine and stereotactic body radiation therapy in locally advanced pancreatic cancer, *Pract Radiat Oncol.* ;6(6):417-424
58. Reeves M. M., C. O. Terranova, J. M. Erickson, J. R. Job, D. S. K. Brookes, N. McCarthy, I. J. Hickman, S. P. Lawler, B. S. Fjeldsoe, G. N. Healy, E. A. H. Winkler, M. Janda, J. L. Veerman, R. S. Ware, J. B. Prins, T. Vos, W. Demark-Wahnefried and E. G. Eakin (2016). Living well after breast cancer randomized controlled trial: Evaluating a telephone-delivered weight loss intervention versus usual care in women following treatment for breast cancer, *BMC Cancer.* 28;16(1):830.
59. Reijneveld J. C., M. J. B. Taphoorn, C. Coens, J. E. C. Bromberg, W. P. Mason, K. Hoang-Xuan, G. Ryan, M. B. Hassel, R. H. Enting, A. A. Brandes, A. Wick, O. Chinot, M. Reni, G. Kantor, B. Thiessen, M. Klein, E. Verger, C. Borchers, P. Hau, M. Back, A. Smits, V. Golfinopoulos, T. Gorlia, A. Bottomley, R. Stupp and B. G. Baumert (2016). Health-related quality of life in patients with high-risk low-grade glioma (EORTC 22033-26033): a randomized, open-label, phase 3 intergroup study*, Lancet Oncol*. ;17(11):1533-1542.
60. Reitenbach E., R. Rödl, G. Gosheger, B. Vogt and F. Schiedel (2016). Deformity correction and extremity lengthening in the lower leg: comparison of clinical outcomes with two external surgical procedures, *Springerplus.* 24;5(1):2003
61. Rodrigues-De-Souza D. P., C. Fernández-De-Las-Peñas, F. J. Martín-Vallejo, J. F. Blanco-Blanco, L. Moro-Gutiérrez and F. Alburquerque-Sendín (2016).Differences in pain perception, health-related quality of life, disability, mood, and sleep between Brazilian and Spanish people with chronic non-specific low back pain, *Braz J Phys Ther*. 16;20(5):412-42
62. Rukstalis D., P. Rashid, W. K. Bogache, R. F. Tutrone, J. Barkin, P. T. Chin, H. H. Woo, A. L. Cantwell, B. E. Cowan and D. M. Bolton (2016). 24-month durability after crossover to the prostatic urethral lift from randomised, blinded sham*, BJU Int*.;118 Suppl 3:14-22
63. Rykov K., I. H. F. Reininga, B. A. S. Knobben, M. S. Sietsma and B. L. E. F. Ten Hav (2016). The design of a randomised controlled trial to evaluate the (cost-) effectiveness of the posterolateral versus the direct anterior approach for THA (POLADA - trial), BMC *Musculoskelet Disord*. 15;17(1):476.
64. Saal D. P., M. J. Overdijk, R. D. Thijs, I. M. Van Vliet and J. G. Van Dijk (2016) Long-term follow-up of psychogenic pseudosyncope, *Neurology.* 22;87(21):2214-2219.
65. Sabadia S. B., R. C. Nolan, K. M. Galetta, K. M. Narayana, J. A. Wilson, P. A. Calabresi, E. M. Frohman, S. L. Galetta and L. J. Balcer (2016). 20/40 or better visual acuity after optic neuritis: Not as good as we once thought? J *Neuroophthalmol.* ;36(4):369-376.
66. Salve V. T. and J. S. Atram (2016).N-Acetylcysteine combined with home based physical activity: Effect on health-related quality of life in stable COPD patients- a randomised controlled trial*, J Clin Diagn Res*. ;10(12): OC16-OC19
67. Shomura M., T. Kagawa, H. Okabe, K. Shiraishi, S. Hirose, Y. Arase, K. Tsuruya, S. Takahira and T. Mine (2016). Longitudinal alterations in health-related quality of life and its impact on the clinical course of patients with advanced hepatocellular carcinoma receiving sorafenib treatment, *BMC Cancer.* 11;16(1):878.
68. Shore S., K. G. Smolderen, K. F. Kennedy, P. G. Jones, S. V. Arnold, D. J. Cohen, J. M. Stolker, Z. Zhao, T. Y. Wang, P. M. Ho and J. A. Spertus (2016). Health Status Outcomes in Patients with Acute Myocardial Infarction after Rehospitalization*, Circ Cardiovasc Qual Outcomes.* ;9(6):777-784
69. Silverberg J. I., K. Hinami, W. E. Trick and D. Cella (2016) Itch in the General Internal Medicine Setting: A Cross-Sectional Study of Prevalence and Quality-of-Life Effects, *Am J Clin Dermatol; 17(6):681-690.*
70. Singh R, Teel C, McGinnis P, Kluding P. (2016) Fatigue in Type 2 Diabetes: impact on Quality of Life and Predictors. *PLoS One.  8;11(11): 0165652.*
71. Smith T. M., C. N. Broomhall and A. R. Crecelius (2016). Physical and psychological effects of a 12-session cancer rehabilitation exercise program, *Clin J Oncol Nurs.* 1;20(6):653-659.
72. Sofianos C. and C. Sofianos (2016) Outcomes of laparoscopic sleeve gastrectomy at a bariatric unit in South Africa, *Ann Med Surg (Lond*). 15; 12:37-42.
73. Soyyiğit Ş., Ö. Aydın, İ. Yılmaz, S. K. Özdemir, V. Ş. Cankorur, C. Atbaşoğlu and G. E. Çelik (2016). Evaluation of drug provocation test–related anxiety in patients with drug hypersensitivity, *Ann Allergy Asthma Immunol*. ;117(3):280-4
74. Stain H. J., S. Bucci, A. L. Baker, V. Carr, R. Emsley, S. Halpin, T. Lewin, U. Schall, V. Clarke, K. Crittenden and M. Startup (2016). A randomized controlled trial of cognitive behaviour therapy versus non-directive reflective listening for young people at ultra-high risk of developing psychosis: The detection and evaluation of psychological therapy (DEPTh) *trial, Schizophrenic Res*. ;176(2-3):212-219.
75. Stewart A. K., M. A. Dimopoulos, T. Masszi, I. Špička, A. Oriol, R. Hájek, L. Rosiñol, D. S. Siegel, R. Niesvizky, A. J. Jakubowiak, J. F. San-Miguel, H. Ludwig, J. Buchanan, K. Cocks, X. Yang, B. Xing, N. Zojwalla, M. Tonda, P. Moreau and A. Palumbo (2016). Health-related quality-of-life results from the open-label, randomized, phase III ASPIRE trial evaluating carfilzomib, lenalidomide, and dexamethasone versus lenalidomide and dexamethasone in patients with relapsed multiple myeloma. [*J Clin Oncol.*](https://www.ncbi.nlm.nih.gov/pubmed/?term=Health-related+quality-of-life+results+from+the+open-label%2C+randomized%2C+phase+III+ASPIRE+trial+evaluating+carfilzomib%2C+lenalidomide%2C+and+dexamethasone+versus+lenalidomide+and+dexamethasone+in+patients+with+relapsed+multiple+myeloma)  10;34(32):3921-3930
76. Stewart G. C., M. M. Kittleson, P. C. Patel, J. A. Cowger, C. B. Patel, M. M. Mountis, F. L. Johnson, M. E. Guglin, J. E. Rame, J. J. Teuteberg and L. W. Stevenson (2016). INTERMACS (Interagency Registry for Mechanically Assisted Circulatory Support) Profiling Identifies Ambulatory Patients at High Risk on Medical Therapy after Hospitalizations for Heart Failure*, Circ Heart Fail.* ;9(11).
77. Strauss S. B., N. Kim, C. A. Branch, M. E. Kahn, M. Kim, R. B. Lipton, J. M. Provataris, H. F. Scholl, M. E. Zimmerman and M. L. Lipton (2016). Bidirectional changes in anisotropy are associated with outcomes in mild traumatic brain injury, AJNR. AmJ*Neuroradiol.* ;37(11):1983-1991
78. Sulaiman I., J. Seheult, E. MacHale, F. Boland, S. M. O'Dwyer, V. Rapcan, S. D'Arcy, B. Cushen, M. Mokoka, I. Killane, S. A. Ryder, R. B. Reilly and R. W. Costello (2016). A method to calculate adherence to inhaled therapy that reflects the changes in clinical features of asthma, *Ann Am Thorac Soc*.;13(11):1894-1903.
79. Tannuri A. C. A., M. A. E. Ferreira, A. L. Mathias and U. Tannuri (2016). Long-Term evaluation of fecal continence and quality of life in patients operated for anorectal malformations*, Rev Assoc Med Bras* (1992) ;62(6):544-552
80. Taylor P. N., I. A. W. Hart, M. S. Khan and D. E. M. Slade-Sharman (2016). Correction of footdrop due to multiple sclerosis using the STIMuSTEP implanted dropped foot stimulator, *Int J MS Care. ;18(5):239-247.*
81. Theeke L. A., J. A. Mallow, J. Moore, A. McBurney, S. Rellick and R. VanGilder (2016). Effectiveness of LISTEN on loneliness, neuroimmunological stress response, psychosocial functioning, quality of life, and physical health measures of chronic illness*, Int J Nurs Sci.* ;3(3):242-251.
82. Theou O., E. C. K. Tan, J. S. Bell, T. Emery, L. Robson, J. E. Morley, K. Rockwood and R. Visvanathan (2016) Frailty Levels in Residential Aged Care Facilities Measured Using the Frailty Index and FRAIL-NH Scale*, J Am Geriatr Soc*. ;64(11): e207-e212.
83. Tomal J., D. McKiernan, S. Guandalini, C. E. Semrad and S. S. Kupfer (2016). Celiac patients' attitudes regarding novel therapies, *Minerva Gastroenterol Dietol*. (2016) ;62(4):275-280
84. Tsuji T., K. Matsudaira, H. Sato and J. Vietri (2016). The impact of depression among chronic low back pain patients in Japan, *BMC Musculoskelet Disord*. 27;17(1):447.
85. Tuite G. F., Y. Homsy, E. G. Polsky, M. A. Reilly, C. M. Carey, S. P. Winesett, L. F. Rodriguez, B. B. Storrs, S. J. Gaskill, L. L. Tetreault, D. G. Martinez and E. K. Amankwah (2016). Urological Outcome of the Xiao Procedure in Children with Myelomeningocele and Lipomyelomeningocele Undergoing Spinal Cord Detethering, *J Urol.* ;196(6):1735-1740.
86. Tušek-Bunc K. and D. Petek (2016). Comorbidities and characteristics of coronary heart disease patients: Their impact on health-related quality of life, *Health Qual Life Outcomes.* 15;14(1):159.
87. Ueberall M. A. and G. H. H. Mueller-Schwefe (2016). Efficacy and tolerability balance of oxycodone/ naloxone and tapentadol in chronic low back pain with a neuropathic component: A blinded end point analysis of randomly selected routine data from 12-week prospective open-label observations, *J Pain Res*. 11; 9:1001-1020.
88. Urbančič J., T. Soklič Košak, K. Jenko, N. Božanić Urbančič, P. Hudoklin, M. Delakorda, A. Juvanec, K. Zupančič Urbančič, J. Vadnjal and D. Gluvajić (2016). Cross-cultural adaptation and validation of nasal obstruction symptom evaluation questionnaire in Slovenian language, *Zdr Varst. 28*;56(1):18-23.
89. Vardy J. L., H. M. Dhillon, G. R. Pond, C. Renton, A. Dodd, H. Zhang, S. J. Clarke and I. F. Tannock (2016). Fatigue in people with localized colorectal cancer who do and do not receive chemotherapy: A longitudinal prospective study*, Ann Oncol*. ;27(9):1761-7.
90. Vasconcelos K. S. S., J. M. D. Dias, M. C. Araújo, A. C. Pinheiro, B. S. Moreira and R. C. Dias (2016). Effects of a progressive resistance exercise program with high-speed component on the physical function of older women with sarcopenic obesity: A randomized controlled trial, *Braz J Phys Ther*. 11;20(5):432-440
91. Verchota G. and K. J. Sawin (2016). Testing components of a self-management theory in adolescents with type 1 diabetes mellitus, *Nurs Res*. ;65(6):487-495
92. Villafañe J. H., C. Pirali, M. Isgrò, C. Vanti, R. Buraschi and S. Negrini (2016). Effects of Action Observation Therapy in Patients Recovering From Total Hip Arthroplasty Arthroplasty: A Prospective Clinical Trial, J *Chiropr Med.* ;15(4):229-234
93. Wakefield C. E., J. E. Fardell, E. L. Doolan, D. Drew, R. De Abreu Lourenco, A. L. Young and R. J. Cohn (2016) Grandparents of children with cancer: Quality of life, medication and hospitalizations*, Pediatr Blood Cancer*. ;64(1):163-171
94. Wang L., X. Xu, Y. Zhang, H. Hao, L. Chen, T. Su, Y. Zhang, W. Ma, Y. Xie, T. Wang, F. Yang, L. He, W. Wang, X. Fu and Y. Ma (2016). A model of health education and management for osteoporosis prevention*, Exp Ther Med*. ;12(6):3797-3805
95. Wang S. H., Y. Z. Ming, P. Y. Lin, J. Y. Wang, H. C. Lin, C. E. Hsieh, Y. L. Hsu and Y. L. Chen (2016). Predictors of diarrhea after hepatectomy and its impact on gastrointestinal quality of life in living donors, *PLoS One.* 18;11(11)
96. Wang X. J., C. M. Wong and A. Chan (2016). Psychometric Properties of the Functional Assessment of Cancer Therapy–Neutropenia in Asian Cancer Patients With Chemotherapy-Induced Neutropenia*, J Pain Symptom Manage*.;52(3):428-36.
97. Warschburger P. and K. Kröller (2016) Loss to follow-up in a randomized controlled trial study for pediatric weight management (EPOC), *BMC Pediatr*. 14;16(1):184.
98. Wei P., J. W. Yang, H. W. Lu, B. Mao, W. L. Yang and J. F. Xu (2016). Combined inhaled corticosteroid and long-acting β2-adrenergic agonist therapy for cystic fibrosis bronchiectasis with airflow limitation: An observational study, *Medicine (Baltimore). ;95(42): e5116.*
99. Wyrwich K. W., S. Krishnan, P. Auguste, J. L. Poon, R. von Maltzahn, R. Yu, G. F. Pierce, B. Mei, J. Mahlangu and S. von Mackensen (2016). Changes in health-related quality of life with treatment of longer-acting clotting factors: results in the A-LONG and B-LONG clinical studies, *Haemophilia.* ;22(6):866-872.
100. Xu M., H. Yu, Y. Chen, J. Xu, J. Zheng and X. Yu (2016). Long-term quality of life in adult patients with strabismus after corrective surgery compared to the general population, *PLoS One. 15;11(11): e0166418.*
101. Yao P., T. Hong, Y. Q. Zhu, H. X. Li, Z. B. Wang, Y. Y. Ding, J. M. Ma and S. N. Pan (2016). Efficacy and safety of continuous radiofrequency thermocoagulation plus pulsed radiofrequency for treatment of V1 trigeminal neuralgia A prospective cohort study, *Medicine (Baltimore)*. ;95(44): e5247.
102. Ünver R., F. Deveci, G. Kırkıl, S. Telo, D. Kaman and M. Kuluöztürk (2016). Serum heat shock protein levels and the relationship of heat shock proteins with various parameters in chronic obstructive pulmonary disease patients, *Turk Thorac* J. ;17(4):153-159.
103. Yoo H. J., H. Kim, H. J. Park, D. S. Kim, Y. S. Ra and H. Y. Shin (2016) Neurocognitive function and health-related quality of life in pediatric Korean survivors of medulloblastoma*, J Korean Med* Sci. ;31(11):1726-1734.
104. Zhang L., Y. Ren and Y. Liu (2016). Comparison of the effects of lobectomy on immunologic function between video-assisted thoracoscopic surgery and traditional open surgery for non-small-cell lung cancer, *Am J Ther.* ;23(6): e1406-e1413.
105. Zickgraf H. F., M. E. Franklin and P. Rozin (2016). Adult picky eaters with symptoms of avoidant/restrictive food intake disorder: Comparable distress and comorbidity but different eating behaviors compared to those with disordered eating symptoms*, J Eat Disord.* 29; 4:26.
